# Modeling Cadmium Exposures in Low- and High-Exposure Areas in Thailand

**DOI:** 10.1289/ehp.1104769

**Published:** 2013-02-22

**Authors:** Soisungwan Satarug, Witaya Swaddiwudhipong, Werawan Ruangyuttikarn, Muneko Nishijo, Patricia Ruiz

**Affiliations:** 1 Centre for Kidney Disease Research, Princess Alexandra Hospital, University of Queensland School of Medicine, Brisbane, Queensland, Australia; 2 Department of Community and Social Medicine, Mae Sot General Hospital, Tak Province, Thailand; 3 Department of Forensic Medicine, Division of Toxicology, Chiang Mai University, Thailand; 4 Department of Public Health, Kanazawa Medical University, Ishikawa, Japan; 5 Computational Toxicology and Methods Development Laboratory, Division of Toxicology and Human Health Sciences, Agency for Toxic Substances and Disease Registry, Centers for Disease Control and Prevention, Atlanta, Georgia, USA

**Keywords:** cadmium, computerized predictive model, diet, exposure source, food, health risk assessment, smoking, tolerable intake, toxicokinetics-based model, urinary threshold

## Abstract

Background: Previous U.S. population modeling studies have reported that urinary cadmium (Cd) excretion patterns differ with age, sex, and dietary exposure; associations between Cd exposures and health outcomes also have differed by age and sex. Therefore, it is important to test models used to estimate Cd exposures across an expanded Cd-exposure range.

Objectives: We estimated relative Cd exposures from both diet and smoking in low- and high-exposure scenarios to provide data for improving risk assessment calculations.

Methods: We used a Cd toxicokinetic–based model to estimate Cd exposures based on urinary Cd levels measured for 399 persons in a low-exposure area (Bangkok) and 6,747 persons in a high-exposure area (Mae Sot) in Thailand.

Results: In Bangkok, we estimated dietary Cd exposures of 50–56 µg/day for males and 21–27 µg/day for females 20–59 years of age who never smoked. In Mae Sot, we estimated dietary Cd exposures of 188–224 µg/day for males and 99–113 µg/day for females 20–59 years of age who never smoked. In Bangkok, we estimated Cd exposures from smoking to be 5.5–20.4 µg/day for male smokers 20–59 years of age. In Mae Sot, we estimated Cd exposures from smoking to be 9.8–26 µg/day for male heavy smokers and 26 µg/day for female heavy smokers.

Conclusion: This study provides estimates of Cd exposures from diet and smoking in low- and high-exposure scenarios. Our findings suggest a relatively small safety margin between the established tolerable Cd reference exposure of 62 µg/day and exposure levels previously associated with evidence of kidney and bone effects in Mae Sot residents, where dietary Cd exposures among women were only 1.6–2.1 times the reference value.

Cadmium (Cd) is a food contaminant that poses a real human health hazard (Järup and Åkesson 2009; Satarug 2012; Satarug et al. 2010). For most people, diet is a primary exposure source (Amzal et al. 2009; Arnich et al. 2012; Louekari et al. 1989; Sand and Becker 2012). For a large portion of the general population, tobacco smoke is a secondary source of Cd (Mortensen et al. 2011). Cd from dietary and smoking exposures can accumulate in various organs and tissues, but the most extensive accumulation occurs in the kidney cortex. Urinary Cd (U-Cd) concentrations correlate more strongly with lung and kidney Cd levels than with age or liver Cd levels (Satarug et al. 2002). In fact, researchers have used U-Cd as a measure of cumulative lifetime exposure (Choudhury et al. 2001; Slob and Krajnc 1993). The World Health Organization (WHO 1989, 1993, 2010) and the European Food Safety Authority (EFSA 2011) consider the kidney to be the most sensitive target organ for Cd effects. Thus WHO and EFSA have established an intake guideline known as a provisional tolerable weekly intake (PTWI).

The PTWI is an estimate of the amount of a chemical with no intended function that can be ingested weekly over a lifetime without appreciable health risk (WHO 1989). The original PTWI for Cd was set at 400–500 µg/person/week (WHO 1989), but it was later revised to 7 µg/kg body weight/week (WHO 1993). In its latest assessment, the WHO adjusted the tolerable intake of Cd to 25 µg/kg body weight/month (62 µg/day for a 70-kg person) (WHO 2010). However, the EFSA (2011) established a tolerable intake of 2.5 µg/kg body weight/week (25 µg/day for a 70-kg person). In addition, the WHO set a U-Cd level of 5.24 µg/g creatinine as a threshold to protect against kidney damage, whereas the EFSA set a U-Cd threshold of 1 µg/g creatinine. Mortensen et al. (2011) reported that Cd exposures in the United States resulted in U-Cd concentrations of > 1 µg/g creatinine in 4.8% of nonsmoking adults and 20.8% of smokers.

In an exposure–response analysis, Järup and Åkesson (2009) noted evidence of adverse effects on kidney and bone at U-Cd concentrations of < 1 µg/g creatinine, raising a concern that the established thresholds might not provide sufficient protection. Further, Ciesielski et al. (2012) reported an association between Cd exposure and parental report of a learning disability in a representative sample of U.S. children enrolled in the National Health and Nutrition Examination Survey (NHANES) that persisted even when children with U-Cd concentrations of > 1 µg/g creatinine were excluded from the analysis. This finding raised a further concern that effects may extend beyond kidney and bone, which other studies appear to corroborate: Cd exposures in representative U.S. adult populations enrolled in NHANES have been reported to be associated with chronic kidney disease and kidney stones (Ferraro et al. 2010, 2011), prediabetes and diabetes (Schwartz et al. 2003), hypertension and cardiovascular disease (Tellez-Plaza et al. 2008, 2010), all-cause mortality (Menke et al. 2009), and overall cancer mortality in both men and women (Adams et al. 2012).

Rather than relying solely on a dietary assessment method known as total diet study (Arnich et al. 2012; Sand and Becker 2012), risk assessments could benefit from an ability to derive Cd exposure levels from population biomonitoring data such as U-Cd excretion. A previous modeling study using data from NHANES III participants 2–70 years of age reported that estimated urinary excretion of Cd from dietary sources was higher in females than males, and higher in the 6- to 11-year-old range than in older age groups (Ruiz et al. 2010b). In the present study, we used Thai population-biomonitoring data to estimate Cd exposure from both diet and smoking as a function of age, sex, and locality. In addition, we compared established tolerable-intake and urinary threshold levels derived for average individuals with estimated intakes and urinary concentrations in population subgroups according to age, sex, and smoking status.

## Materials and Methods

*Sample populations*. To represent a group with chronic high-dose exposure, we assembled a sample population of 6,747 residents from 12 rural farming villages in Thailand’s Mae Sot District, Tak Province. These villages are in an area where environmental Cd contamination has occurred (Swaddiwudhipong et al. 2007, 2010a). Associations of urine Cd concentrations with hypertension and markers of kidney and bone disease in Mae Sot residents have been reported previously (Honda et al. 2010; Limpatanachote et al. 2010; Swaddiwudhipong et al. 2010b; Teeyakasem et al. 2007). The Mae Sot Hospital ethical committee approved our study, and the informed participants verbally consented to participate. After obtaining the participants’ informed consent, we also obtained information from a low-exposure group of 399 apparently healthy persons who had no exposure to Cd in the workplace and who lived in Bangkok at the time of the study (Satarug et al. 2004a). The institutional ethical committee, Chulalongkorn University Hospital, approved the Bangkok protocol. The Bangkok sample provided comparative information as a low-exposure group.

*Exposure assessment*. For the Bangkok group, U-Cd concentrations were determined with inductively coupled plasma/mass spectrometry, calibrated with multi-element standards (EM Science; EM Industries Inc., Whitehouse Station, NJ, USA) (Satarug et al. 2004a). Quality assurance and control were conducted with simultaneous analysis of samples of the reference urine Lyphochek® (Bio-Rad, Gladesville, New South Wales, Australia), which contained low- and high-range Cd levels. A coefficient of variation value of 2.5% was obtained for Cd in the reference urine. Cd concentrations of urine samples < 0.05 µg/L limit of detection (LOD) were assigned the LOD divided by the square root of 2. The automated system at the Chulalongkorn University Hospital, Bangkok, Thailand, was used to determine urinary creatinine concentrations based on Jaffe’s reaction. For the Mae Sot group, U-Cd concentrations were determined with an atomic absorption spectrometer (Varian Model AA280Z; Varian Inc., Palo Alto, CA, USA) in the Thailand Ministry of Public Health’s laboratory (Swaddiwudhipong et al. 2010a). Quality assurance and control were conducted with reference urine certified by the German External Quality Assessment Scheme. Urinary creatinine concentrations based on Jaffe’s reaction were determined using an autoanalyzer (Konelab 30; Thermo Electron Corp., Vantaa, Finland).

*Computerized simulation model of Cd toxicokinetics*. The computerized predictive model used in this study is available in the Agency for Toxic Substances and Disease Registry’s (ATSDR) Computational Toxicology Laboratory physiologically based pharmacokinetic (PBPK) toolkit (Ruiz et al. 2010a, 2010b, 2011). The predictive model consists of a series of models recoded in one simulation language: Berkeley-Madonna software (version 8.01 for Windows; Kagi Shareware, Berkeley, CA, USA) (Ruiz et al. 2010a, 2010b, 2011). The model was based on the Kjellström and Nordberg (1978) Cd toxicokinetics model that Choudhury et al. (2001) and Diamond et al. (2003) later modified. The model was validated with data from the Fourth National Report on Human Exposure to Environmental Chemicals as detailed elsewhere by Ruiz et al. (2010a). Briefly, we calculated the percent median absolute performance error (MAPE%) based on estimates of performance error (PE), used root median square performance error (RMSPE%) to estimate the prediction’s accuracy, and used a sensitivity ratio (SR) approach to assess the robustness of each recoded model (Ruiz et al. 2010a). The model’s ability to simulate U-Cd measured for nonsmoking populations in NHANES III, using U.S. dietary Cd exposures as the input dataset, has been reported together with complete details on the model structures and parameters (Ruiz et al. 2010b).

*Estimates of dietary Cd exposures*. For oral-route exposure, our initial model input was the amount of dietary Cd ingested per day (micrograms per day) estimated for age- and sex-specific groups as reported by Choudhury et al. (2001). We iteratively increased the estimated dietary intakes until geometric creatinine-adjusted U-Cd values (micrograms per gram creatinine) predicted by the model replicated as closely as possible the geometric mean (GM) U-Cd values measured in Bangkok and Mae Sot study participants who never smoked. In this way we obtained the dietary Cd exposure estimates for the age and sex-specific groups in both Bangkok and in Mae Sot.

*Estimates of Cd exposures from smoking*. Based on tobacco Cd content data reported for various Asian cigarette brands (O’Connor et al. 2010), we assumed that for every pack of cigarettes, Bangkok smokers inhaled 5.5 µg of Cd. We assumed Mae Sot smokers inhaled 6.5 µg Cd/pack of cigarettes, given the potential increase in pulmonary absorption due to anemia (Swaddiwudhipong et al. 2007), which can promote Cd uptake (Satarug et al. 2002, 2004b). To estimate overall Cd exposure (i.e., oral plus inhalation) for smokers, we used the model-based age-, sex-, and population-specific estimates of GM dietary-Cd exposure derived for those who never smoked and the number of packs smoked per day reported by participants, as the initial model input for each group of smokers. We then obtained model-based estimates of the number of packs of cigarettes smoked per day by iteratively increasing the number of packs smoked until the GM U-Cd values predicted by the model replicated GM U-Cd values measured in the smokers. To estimate Cd exposure from smoking, we multiplied number of packs smoked per day by 5.5 and 6.5 µg for smokers in Bangkok and Mae Sot, respectively.

*Statistical analysis*. Statistical analysis was performed with the SPSS statistical package for Windows (version 16; IBM, New York, NY, USA). The Kolmogorov–Smirnov goodness-of-fit test was used to evaluate conformity to normal distributions of measured and base-10 logarithmically transformed data. Differences between the Bangkok and Mae Sot populations were evaluated using Student’s *t*-test for normally distributed variables, and the Mann–Whitney *U* test for variables that were not normally distributed. Differences in logarithmically transformed U-Cd among ≥ 3 groups (e.g., when stratified by age and smoking habits) were estimated using one-way analysis of variance, followed by the Dunnett post hoc test. Results were considered statistically significant if *p* ≤ 0.05.

## Results

Subjects from Bangkok were, on average, in their early-to-mid-30s in age (years), whereas the Mae Sot subjects were in their mid-40s (Table 1). The prevalence of smoking was high in Mae Sot compared with Bangkok, and in males compared with females. About 70% of Mae Sot males smoked, and > 40% were classified as heavy smokers (26–80 cigarettes/day). In Bangkok, about 40% of males smoked, and none were classified as heavy smokers. Bangkok had no female smokers, but 4.4% and 11% of Mae Sot females were light-to-moderate and heavy smokers, respectively.

**Table 1:** Characteristics of Bangkok and Mae Sot groups.

Variable	Bangkok		Mae Sot	
	Males	Females	Males	Females
No. of persons	199	200	3,021	3,726
Age (years, mean ± SD)	32.9 ± 8.8*	36.9 ± 10	47.7 ± 16.6*	46.4 ± 15.2
Smoking status (%)
Never smokers	59.3	100	27.2	77.2
Former smokers	0	0	20.9	7.4
Current smokers
Light to moderate	40.7	0	9.2	4.4
Heavy	0	0	42.7	11.0
U‑Cd (µg/g creatinine, GM ± SD)	0.40 ± 0.46*	0.50 ± 0.46	1.65 ± 2.40*	2.10 ± 2.91
U‑Cd ≥ 1 µg/g creatinine (%)
Never smokers	5.9	22.5	58.0	76.0
Former and current smokers	13.6	0	77.2	94.9
U‑Cd ≥ 5 µg/g creatinine (%)
Never smokers	0	0	4.9	12
Former and current smokers	0	0	10.4	26.5
Light-to-moderate smokers, 10–25 cigarettes/day; heavy smokers, 26–80 cigarettes/day. *p < 0.001 compared with females in each population.				

As expected, there were pronounced differences in measured U-Cd between the two communities. GM values were 0.40 and 1.65 µg/g creatinine in Bangkok and Mae Sot males, respectively, and 0.50 and 2.10 µg/g creatinine in Bangkok and Mae Sot females, with significantly higher mean values in females compared with males in both communities (both *p* < 0.001). Among never smokers, 22.5% and 5.9% of females and males in Bangkok, and 76% and 58% in Mae Sot, respectively, had measured U-Cd concentrations of ≥ 1 µg/g creatinine. In Mae Sot, 12% and 4.9% of females and males who never smoked had U-Cd concentrations of ≥ 5 µg/g creatinine, compared with 26.5% and 10.4% of females and males who were current or former smokers.

Measured GM U-Cd values are shown according to age, sex, smoking status, and location in Table 2. Overall, measured U-Cd values increased with age, and were higher in females than males, and in smokers compared with nonsmokers. In the Bangkok group, the GM values for U-Cd in male smokers in 20- to 39-year-old (0.41 µg/g creatinine) and the 40- to 59-year-old (0.92 µg/g creatinine) age groups were, respectively, 1.3 times and 1.9 times greater than in males in the same age groups who never smoked (0.33 and 0.49 µg/g creatinine). Similarly, in Mae Sot, the measured U-Cd for heavy smokers who were 40–59 years of age and those ≥ 60 years were 1.4 times and 1.5 times greater than males in the same age groups who never smoked. The U-Cd for female heavy smokers in the 40- to 59-year-old and ≥ 60-year-old groups was 1.6 times and 1.3 times greater than for females in the same age groups who never smoked. In Mae Sot, measured U-Cd values in older males and females who were heavy smokers were significantly higher (*p* > 0.001 to < 0.002) than in corresponding male and female never smokers.

**Table 2:** Measured U‑Cd (µg/g creatinine) by age (years), sex, cigarette smoking status, and locality.

Smoking status	Bangkok						Mae Sot							
	Males			Females			Males				Females			
	13–19	20–39	40–59	13–19	20–39	40–59	13–19	20–39	40–59	≥ 60	13–19	20–39	40–59	≥ 60
Never smokers	0.30 (0.10)	0.33 (0.36)	0.49 (0.38)	0.35 (0.22)	0.48 (0.43)	0.54 (0.51)	0.70 (0.84)	1.04 (1.61)	1.50 (1.98)	1.85 (2.47)	0.95 (1.36)	1.49 (2.26)	2.08 (2.82)	3.07 (3.79)
Smokers
Former smokers	—	—	—	—	—	—	0.45 (0.46)	0.99 1.54)	1.78 (3.11)	2.45 (3.04)	1.93 (0.36)	1.74 (1.24)	2.89 (2.78)	3.70 (3.09)
Current
Light/moderate smokers	0.28 (0.32)	0.41 (0.41)	0.92 (0.93)	—	—	—	0.72 (0.54)	1.00 (1.15)	2.07 (1.78)	2.55 (2.53)	—	2.10 (1.48)	2.75 (2.26)	3.13 (3.73)
Heavy smokers	—	—	—	—	—	—	0.79 (0.61)	1.17 (1.51)	2.12* (2.54)	2.84* (4.46)	0.73 (0.08)	1.91 (1.59)	3.41** (2.66)	3.92** (3.46)
Values are GM (SD) for measured U‑Cd. —, ≤ 5 subjects; light-to-moderate smokers, 10–25 cigarettes/day; heavy smokers, 26–80 cigarettes/day. *p < 0.001 compared with never smokers of the same age, sex, and location. **p > 0.001 to < 0.002 compared with never smokers of the same age, sex, and location.														

Age- and sex-specific model–based estimates of dietary Cd exposure among never smokers in Bangkok and Mae Sot are shown in Table 3. In Bangkok, dietary Cd exposures of 56 and 50 µg/day were estimated for males in the 20- to 39-year-old and 40-to 59-year-old groups, respectively, who never smoked. Dietary Cd exposures of 27 and 21 µg/day were estimated for the 20- to 39-year-old and 40- to 59-year-old females, respectively, who never smoked. Much higher dietary Cd exposures were estimated for all age- and sex-specific groups in Mae Sot compared with their counterpart groups in Bangkok. For Mae Sot, estimates of dietary Cd exposures were 234, 224, 188, and 167 µg/day, respectively, for males who never smoked, ages 13–19, 20–39, 40–59, and ≥ 60 years. The corresponding dietary Cd exposures, estimated for females who never smoked, were 132, 113, 99, and 118 µg/day, respectively.

**Table 3:** Estimates of dietary Cd exposures from the toxicokinetic model for never smokers by age, sex, and locality.

Age group (years)	Bangkok				Mae Sot			
	Males		Females		Males		Females	
	n	Diet Cd (µg/day)	n	Diet Cd (µg/day)	n	Diet Cd (µg/day)	n	Diet Cd (µg/day)
13–19	—	—	—	—	152	234	221	132
20–39	85	56	117	27	191	224	903	113
40–59	31	50	81	21	327	188	1,394	99
≥ 60	—	—	—	—	151	167	358	118
— , Not included because n < 5.								

Model-based estimates of numbers of packs of cigarettes smoked per day according to age-, sex-, and location among smokers are shown in Table 4. Estimates for current, light-to-moderate male smokers in Bangkok were 1 and 3.7 packs/day for 20-to 39-year-old and 40- to 59-year-old groups, respectively, resulting in Cd exposure estimates of 5.5 and 20.4 µg/day. For male heavy smokers in Mae Sot, estimates were 1.5, 1.5, 3, and 4 packs/day for the 13- to 19-, 20- to 39-, 40- to 59-, and ≥ 60-year-old groups, respectively, resulting in estimated Cd exposures of 9.8, 9.8, 19.5, and 26 µg/day, respectively. For female heavy smokers in Mae Sot, the estimated smoking rate was 4 packs/day for all age groups, resulting in estimated Cd exposures of 26 µg/day (data not shown).

**Table 4:** Estimates of number of packs of cigarettes smoked per day from the toxicokinetic model for current smokers by age, sex, and locality

Age group (years)	Bangkok				Mae Sot			
	Males		Females		Males		Females	
	n	Packs/day	n	Packs/day	n	Packs/day	n	Packs/day
13–19	—	—	—	—	55	1.5	—	—
20–39	65	1.0	—	—	296	1.5	38	4
40–59	12	3.7	—	—	663	3.0	207	4
≥ 60	—	—	—	—	277	4.0	162	4
— , Not included because n < 5. For Bangkok, smoking was estimated for current light-to-moderate smokers only. For Mae Sot, smoking was estimated for current heavy smokers only.								

## Discussion

*Exposure levels and exposure sources*. Among subjects in this study, age, sex, smoking, and locality were sources of U-Cd variability. A comparison of observed U-Cd data from males and females who never smoked in the 20- to 39-year-old and 40- to 59-year-old groups in Mae Sot with respective counterparts in Bangkok indicated overall Cd exposure levels in Mae Sot to be 3- to 3.8-times greater than in Bangkok. Evidence supporting diet as a major source of high Cd exposures in Mae Sot comes from a previous report indicating that Cd levels in most staple food (rice) samples from Mae Sot were above the permissible limit of 0.2 mg/kg (Swaddiwudhipong et al. 2007). In another report, Cd content in 524 rice samples was 0.05–7.7 mg/kg, with over 90% of samples > 0.2 mg/kg (Simmons et al. 2005). Further, U-Cd levels were higher among persons who consumed locally grown rice compared with those who consumed rice purchased from other areas (Swaddiwudhipong et al. 2007). U-Cd levels were also higher in Mae Sot residents who regularly consumed water from local wells compared with those who did not (Honda et al. 2010). Vegetables and other food crops grown locally in Mae Sot could also be a source of Cd exposure, as was the case with soybeans, all of which samples contained Cd above 0.2 mg/kg (Swaddiwudhipong et al. 2007).

*Exposure from diet*. In this study, estimated dietary Cd exposures were greater in men than in women in both communities. This may be at least partly due to higher food intake in men: An assumption of the Cd-toxicokinetics model used to estimate intakes was that daily Cd exposures increase with caloric intake (Kjellström and Nordberg 1978). Estimated dietary intakes of Cd for all age groups based on 3-day food records were higher in men than women in a previous study of 1,348 persons in Finland (13–17 µg/day in men compared with 12–13 µg/day in women) (Louekari et al. 1989) and were higher for men than women according to model-based predictions for U.S. populations (15–22.4 µg/day for men compared with 13.5–16.5 µg/day for women) (Ruiz et al. 2010b). In contrast, Sand and Becker (2012) observed no sex differences in dietary Cd intakes based on 7-day food records from a survey of > 1,200 adults in Sweden and reported that dietary Cd exposure for an average 70-kg consumer was 10 µg/day, with 40–50% of dietary Cd coming from staple foods (potatoes and wheat), whereas for high consumers (dietary Cd exposure > 95th percentile) estimated dietary exposure was 22 µg/day, with additional Cd coming from seafood and spinach. Tellez-Plaza et al. (2012) reported a decrease in mean values of U-Cd in the United States from 1988–2008 that may have resulted from reductions in smoking exposure. This suggests diet remains the main Cd exposure source in the U.S. population; that Cd in human urine samples originates from diet and smoking is well established (Satarug 2012). Arnich et al. (2012) reported that the Cd concentrations measured in prepared food samples representing the diet of the French population were higher in total diet samples collected in 2007–2009 compared with samples collected in 2000–2004.

In the present study, estimated dietary Cd exposures for the Bangkok population were 21–27 µg/day and 50–56 µg/day for 20- to 59-year-old women and men, respectively (Table 3). The female dietary Cd exposure data were consistent with GM values of 24.7 and 35.7 µg/day based on Cd measured in duplicate diet samples from Japanese women (Ikeda et al. 2000; Shimbo et al. 2000). Reeves and Vanderpool (1997) estimated a daily intake of 36 µg of Cd among frequent consumers of sunflower kernels based on Cd measured in duplicate food samples. Based on telephone interviews, Copes et al. (2008) estimated Cd exposure from oysters among oyster growers to be 24.8 µg/day. Vahter et al. (1996) analyzed Cd content of duplicate diets collected in 4 consecutive days, and they reported dietary Cd exposure to be 11 µg/day for Swedish women consuming a mixed diet and 28 µg/day for those consuming a diet high in shellfish. Based on Cd content of duplicate diets and dietary records, Berglund et al. (1994) reported the average dietary Cd exposure ranged from 5–38 µg/day among 57 nonobese, 20- to 50-year-old Swedish women.

By comparison, for men and women 20–59 years of age in the Mae Sot group, estimated dietary Cd exposures were 188–224 and 99–113 µg/day, respectively (Table 3), consistent with an estimate of > 200 µg/day for dietary exposure in a Japanese population living in a Cd-contaminated area (Iwata et al. 1993).

Our model may have overestimated dietary Cd exposure because it did not account for water as an additional exposure source. In addition, model estimates do not reflect potential variation in Cd accumulation and urinary excretion (Slob and Krajnc 1993) related to exposure variability, age-related kidney degeneration, changes in exposure over time due to lifestyle changes, and the influence of iron, calcium, and zinc intakes on Cd absorption and excretion. However, Amzal et al. (2009) showed that the model performed well when daily exposure is relatively constant because staple foods are the main exposure source. Satarug et al. (2004b) reported that U-Cd concentrations were 3–4 times higher among women who had low iron stores compared with women of similar age whose iron stores were normal. Kippler et al. (2007) reported higher U-Cd in women who had low iron stores but adequate zinc status compared with women who had both low iron and zinc status. Julin et al. (2011) reported that a model performed well when age, body weight, and body iron store status were incorporated.

*Exposure from smoking*. We estimated that in Bangkok, male smokers 20–39 and 40–59 years of age smoked 1 and 3.7 packs/day, respectively, resulting in estimated Cd exposures of 5.5 and 20.4 µg/day, assuming an inhaled Cd dose of 5.5 µg/pack. However, average smoking rates reported by Bangkok males ages 20–39 and 40–59 years were only 0.42 and 0.7 packs/day, respectively. Based on the model’s assumption of inhaled Cd at 6.5 µg/pack, estimates of smoking rates in Mae Sot males of 1.5–4 packs/day were comparable to the numbers of packs per day reported by the study participants (2–4 packs). Differences between model-based estimates and estimates based on self-reported smoking could include underreporting of smoking, inhalation of Cd in air (e.g., from passive smoking), and variability in tobacco Cd levels. Pappas et al. (2007) reported that Cd in mainstream smoke varied from 0.02–0.35 µg/cigarette, while O’Connor et al. (2010) reported that Cd ranged from 1–2.7 µg/cigarette in Chinese cigarette brands, which is 3 times higher than Canadian brands. In addition, it has been estimated that 2.7 µg/day Cd could be inhaled from ambient air if 75% of the population smoked an average of 24 cigarettes/day (Kjellström and Nordberg 1978).

Bangkok smokers were light-to-moderate smokers with lower dietary background exposures, in contrast with Mae Sot smokers, most of whom were heavy smokers with high background exposures. Background Cd exposures alone can cause kidney damage and loss of renal proximal epithelial cells, resulting in high U-Cd. Further, nicotine can also cause kidney damage among smokers (Hallan and Orth 2011; Jaimes et al. 2007). Our model does not account for nicotine-induced kidney damage.

*Implications for risk assessment of dietary Cd exposure*. Risk assessments have established tolerable intake and urinary threshold levels to protect against kidney damage. The WHO (2010) set the tolerable intake at 62 µg/day for a 70-kg person, and the urinary threshold at 5.24 µg/g creatinine. The EFSA (2011) set its tolerable intake at 25 µg/day for a 70-kg person and its urinary threshold at 1 µg/g creatinine. Our model-based estimates of dietary Cd for Bangkok never smokers ages 20–39 and 40–60 years were 27 and 21 µg/day for females, and 56 and 50 µg/day for males, respectively. These dietary Cd exposure estimates were within the WHO-established tolerable intakes, but Cd exposures for never smokers in Mae Sot (132, 113, 99, and 118 µg/day for females and 234, 224, 188, and 167 µg/day for males) all were above the reference tolerable intake levels. In Mae Sot, the dietary Cd exposures in women who never smoked were 1.6–2.1 times greater than the WHO tolerable intake, whereas the exposures among male counterparts were 2.7–3.8 times greater.

These estimates suggest relatively small differences between the WHO tolerable intake level and exposure levels that have been associated with evidence of adverse effects on kidney and bone in cross-sectional studies of Mae Sot residents (Honda et al. 2010; Limpatanachote et al. 2010; Swaddiwudhipong et al. 2010b; Teeyakasem et al. 2007). In addition, we estimated that dietary Cd exposures of ≥ 62 µg/day (the WHO tolerable intake level) were associated with U-Cd concentrations of 0.70–1.85 µg/g creatinine in men and 0.95–3.07 µg/g creatinine in women, which are substantially lower than the WHO urinary threshold of 5.24 µg/g creatinine. Therefore, we conclude that to provide adequate protection, U-Cd threshold should be < 1 µg/g creatinine and dietary exposure should be < 62 µg/day. In Bangkok, dietary Cd intakes were associated with U-Cd concentrations of ≥ 1 µg/g creatinine in 22.5% of women who never smoked. NHANES data suggest that 4.8% of nonsmokers and 20.8% of smokers in the United States have U-Cd concentrations of ≥ 1 µg/g creatinine, although the population mean was estimated to be < 0.5 µg/g creatinine (Mortensen et al. 2011; Tellez-Plaza et al. 2012).

*Strengths and limitations*. The main strengths of this study include the large population samples of men and women with moderate-to-high U-Cd levels, a wide age range (13–92 years) among participants, and homogeneous exposure sources (i.e., none were occupationally exposed). U-Cd levels, which are a marker of cumulative lifetime Cd exposure, increased steadily with age in both males and females, reaching a plateau in groups who were ≥ 60 years of age. We know of no other studies in any Cd-contaminated areas that cover both men and women in such large numbers and with as wide an age range.

A limitation of the model-based transformation of data on internal dose (U-Cd concentrations) to external dose levels (intake via diet and smoking) is that the model parameters (body weight, organ weight, and average consumption of different foods) are based on characteristics of an average consumer, and thus does not account for at-risk subpopulations such as females and smokers. A lack of data on Cd in ambient air was also a limitation, although a previous estimate of Cd exposure from passive smoking (2.7 µg/day where 75% of population smoked an average of 24 cigarettes/day) (Kjellström and Nordberg 1978) was substantially lower than our estimates of dietary Cd exposure. Limited smoking data in some groups was a further limitation, as was the assumption that smokers had the same dietary Cd exposures as those who never smoked.

## Conclusions

Our computerized simulation model could be useful in health risk assessments because it enables the use of measured U-Cd data to estimate Cd exposures from diet and smoking according to age and sex. For example, we estimated that Cd exposure from dietary sources in Mae Sot females who never smoked was 1.6–2.1 times greater than the WHO tolerable intake guideline. This suggests that there may be a relatively small safety margin between the reference level and exposure levels previously associated with adverse health effects in cross-sectional studies of Mae Sot residents. The safety margin may be even lower in smokers, whose Cd exposures were approximately 1.3–1.9 times higher than in never smokers. Smoking exposures thus should to be included in overall risk assessment calculations. Data from the present study also may be relevant in developing risk assessment approaches to improve the protection of subpopulations at particular risk by determining daily tolerable exposures based on both sex and smoking status.

The model on which the WHO PTWI was based did not include an uncertainty factor to account for smoking or other nondietary routes of exposure, or for factors such as age and sex (WHO 1993). Our findings suggest the need for a tolerable intake estimate that includes an uncertainty factor of ≥ 10 to protect against kidney damage in > 95% of the population, including female smokers, a subpopulation that may be at especially high risk.
